# Deep learning driven, image-based phenotyping of seed processing efficiency in sainfoin (*Onobrychis viciifolia*)

**DOI:** 10.3389/fpls.2025.1655350

**Published:** 2025-09-23

**Authors:** Bo Meyering, Spencer Barriball, Brandon Schlautman

**Affiliations:** Perennial Legumes Program, The Land Institute, Salina, Kansas, KS, United States

**Keywords:** deep learning, perennial legume breeding, power analysis, seed imaging, small object detection

## Abstract

**Introduction:**

Sainfoin (*Onobrychis* spp.) is a perennial legume traditionally cultivated as a forage crop and is now emerging as a promising candidate for development as a perennial grain legume. Despite its potential, no research has addressed the breeding of sainfoin varieties with superior grain processing properties.

**Methods:**

We conducted a multifactorial experiment to evaluate the depodding and dehulling efficiency of five commercially available sainfoin varieties. Seeds were processed using two different methods (belt thresher and impact dehuller) across five sample sizes. A pre-trained Faster R-CNN (Region-based Convolutional Neural Network) object detection model was fine-tuned to identify intact pods, whole seeds, and split seeds from images of the processed mixtures. These predictions were used to calculate processing efficiency (PE) for each variety. A comprehensive power analysis was performed to determine the minimum sample size of sainfoin pods required to detect differences in PE with high statistical power.

**Results:**

We observed strong varietal differences in PE, as well as clear effects of the processing method. Belt threshing produced mixtures with more intact pods, while the impact dehuller generated a higher proportion of split seeds. Increasing sample size led to more intact pods across all varieties and methods, and notably decreased seed proportion in belt-threshed samples. Statistical modeling combined with object detection outputs revealed that a minimum of 2 g of pods is required to reliably detect an absolute proportional difference of 0.25 in PE between two breeding lines with 80% power.

**Discussion:**

Our findings demonstrate that sainfoin varieties differ significantly in processing efficiency and that processing outcomes depend strongly on both method and sample size. Integrating deep learning–based phenotyping with robust statistical design enables efficient evaluation of processing traits and provides actionable guidelines for breeding programs. While deep learning models offer powerful, cost-effective tools for plant phenotyping, their outputs must be paired with rigorous statistical design to yield reliable and actionable insights for crop improvement.

## Introduction

Sainfoin (*Onobrychis viciifolia*) is a perennial forage legume which originated in the near east, and has been under continuous cultivation as a forage crop across Europe and the Middle East for over 1000 years (Poudel et al., 2023). When grown as a forage crop, sainfoin has many benefits, some of which include anthelminthic and bloat reduction properties in ruminants (Sottie et al., 2014; Desrues et al., 2016), benefits to pollinators (Sheppard et al., 2019; Fratianni et al., 2024), and the potential to reduce greenhouse gas emissions (Sakhraoui et al., 2024). In addition, sainfoin has the potential to become a perennial, temperate zoned pulse crop due to its ease of cultivation and grain nutritional qualities comparable with those of conventional, annual pulses (Craine et al., 2023; Craine et al., 2024b). Recent studies have further highlighted sainfoin’s potential as a dual-use, perennial grain by demonstrating its favorable amino acid profile and absence of detectable mycotoxins, supporting its viability as a safe and nutritious pulse crop (Craine et al., 2024c; Craine et al., 2024a).

Grains from most annual pulse crops must be depodded and dehulled from the seed coat before they are used as a food product or agricultural commodity due to the presence of bitter compounds and high polyphenol content (Singh, 1995). Considering this, developing lines with good processing and seed dehulling properties are important goals for grain legume breeding programs (Wang, 2008; Oomah et al., 2010). Dehulling can be a labor-intensive process and varies widely between crops. Goyal, Vishwakarma, and Wanjari (Goyal et al., 2008) found that a tempering pigeon peas to 10.1% moisture and applying a pretreatment of mustard oil greatly improved the dehulling efficiency (*DE*, ease of removing the hull from whole seed to yield split seeds). Other studies in pigeon peas and mung beans report that combinations of steam treatment, drying, and tempering result in high *DE* (Opoku et al., 2003). Sreerama, Sashikala, and Pratape (Sreerama et al., 2009) pretreated gram species (*Vigna mungo*) with protease and xylanase prior to dehulling, and found benefits in *DE* when compared to oil-treated controls. Lentils are generally soaked, dried, and then tempered back to a specific moisture content before mechanical dehulling (Erskine et al., 1991a; Erskine et al., 1991b; Wang, 2005).

However, to date no research papers have systematically focused on specific methodologies related to depodding and dehulling sainfoin, nor evaluated the *DE* of currently available commercial cultivars. In part, this is because, as a novel perennial grain legume, there is an open question as to whether the most desirable end product is whole seed (WS, i.e. depodded whole seeds) or split seed (SS, i.e. depodded and dehulled seeds). Tarasenko, Butina, and Gerasimenko (Tarasenko et al., 2015) found that flour made from WS had high protein and fiber content with lower fat content, results that were later corroborated in nutritional studies of WS by Craine et al (Craine et al., 2023). A comprehensive analysis of heavy metals and toxins in WS revealed no analytes that pose any threat to human consumption (Craine et al., 2024c). In addition, animal feeding studies using both WS and SS found no difference between in piglet weight gain and conversion ratios (Baldinger et al., 2016), nor were there differences in protein digestibility between sainfoin and other leguminous feedstuff (Kortelainen et al., 2014). This suggests that either WS, SS, or both could potentially be target end-products after processing.

To establish sainfoin as a viable grain crop, improving seed processing traits such as free threshing (ease of seed separation from pods) and dehulling efficiency (*DE*) is critical, as these characteristics directly impact grain quality and food functionality, as shown in other legumes (Ghavidel and Prakash, 2006; Oghbaei and Prakash, 2016). However, evaluating these traits in sainfoin is challenging due to a lack of methodological precedents. In most legumes, *DE* is influenced not only by genetic and environmental factors (Wang, 2008), but also by the type of processing machinery used, including abrasive (Reichert et al., 1984) and centrifugal impact methods (Hlavangwani et al., 2025). Without established benchmarks or standardized methods, defining and improving these traits demands innovative approaches.

Image-based phenotyping offers a promising solution by using computer vision and machine learning to generate high-resolution, reproducible data across breeding lines. While traditional image analysis methods like Fourier elliptical descriptors (Iwata et al., 2010; Schlautman et al., 2020) and morphological operations (Tanabata et al., 2012; Zhang et al., 2018) have been used for trait extraction in other crops, they often suffer from sensitivity to lighting, background conditions, and object alignment (Williams et al., 2013), which limits scalability. In contrast, modern deep learning–based pipelines offer greater robustness and throughput for phenotyping novel grain crops like sainfoin. These tools have already demonstrated utility in legumes for tasks such as species identification (Koklu and Ozkan, 2020; Taheri-Garavand et al., 2021; Rimi et al., 2022), seed detection (Ouf, 2023), and trait extraction using semantic segmentation models (Morales et al., 2024). Such pipelines can automate classification of seed components—intact pods (IP), whole seeds (WS), and split seeds (SS)—to estimate seed processing traits efficiently across genotypes and processing conditions and can help uncover genetic and environmental influences to support targeted breeding strategies.

Yet even with these advanced image-based methods, the reliability and scientific value of phenotyping pipelines ultimately depend on proper experimental design and statistical validation. However, many researchers fail to address proper validation of their image analysis techniques, as Lobet (2017) rightly points out. Few perform proper power analyses before the experiment is completed (Thomas, 1997), let alone after, leading to published results across the sciences that are often misleading or outright false (Ioannidis, 2005). Proper power analysis is essential to ensure experiments are statistically robust. In low-yielding crops or where breeding line seed availability is limited, power analysis guides optimal sample sizes to detect meaningful trait differences between two single lines. This prevents misinterpretation due to under-powered tests and ensures confidence in phenotypic estimates for breeding applications.

The goal of the present study was to 1) train a Faster R-CNN model on processed mixtures to detect and classify IP, WS, and SS, 2) derive a new generalized metric, designated as processing efficiency (*PE*), from the model predictions, 3) evaluate the effects of processing method, variety, and sample size on seed counts and *PE* within a factorial experimental design, 4) perform a power analysis to determine the minimum sample size required to reliably estimate *PE* for future breeding applications, and 5) demonstrate that image-based phenotyping combined with statistical rigor can support the development of sainfoin as a viable perennial grain crop.

## Methods and materials

### Seed material

Sainfoin seed pods, i.e. sainfoin fruit, (an individual seed inside a pod) of five different commercially available varieties were acquired in bulk from seed growers and seed companies: ‘AAC Mountainview’ (Preferred Alfalfa Genetics, Story City, IA), ‘Rocky Mountain Remont’ (Montana Seeds, Inc.; Conrad, MT), ‘Delaney’, ‘Eski’, and ‘Shoshone’ (Alaska Ranch, Twin Bridges, MT). We processed seed pods as received - no additional sorting or quality control was performed on individual pod samples used in this study.

### Experimental design and processing

We designed a full factorial, completely randomized experimental design with the factors ‘variety’, ‘sample-size’, and ‘processing-method’. Each combination of ‘variety’ x ‘sample-size’ x ‘processing-method’ was replicated ten times resulting in a total of 500 individual samples. Seed pods of each variety were randomly sampled in quantities from one to five grams in one-gram increments and weighed on an analytical balance to the nearest 0.0001g to record the true weight of the sample (‘legume_fruit_pod_mass_g’). These sample sizes were chosen to reflect pod sample masses that could feasibly harvested from a single plant breeding line. Samples were placed into coin envelopes, assigned a random ID and a processing method, and stored in our climate-controlled seed vault at 8-10°C and 40-50% relative humidity until they were processed.

We evaluated two different processing methods. Seed pods were processed either by a belt thresher (BT14 Single Plant Belt Thresher, Almaco, IA) which removes seeds from the pod carpel while minimizing damage to the seed, or by an impact type dehuller (LT-15 Laboratory Thresher, Haldrup USA, Inc., IN) which uses rubber impactors in a concave drum to remove seeds from the seed pods. Pod samples processed by the belt thresher were passed through a total of three times. We found that a single pass with our small-scale belt thresher did not adequately remove seeds from the pod carpels; repeated passes ensured more thorough depodding of the samples. We note that this requirement may be specific to our instrument, and processing needs may differ for larger threshers or those produced by other manufacturers. Samples processed by the impact dehuller were passed through one time and processed for a total of 35 sec at speed 9. Once processed, the resultant mixture of IP, WS, and SS was weighed again on an analytical scale, *sans* empty pods and other dehulling debris, and was recorded as ‘processed_mixture_mass_g’. All 500 seed pod samples were processed in this fashion.

### Seed imaging

**Figure 1:**
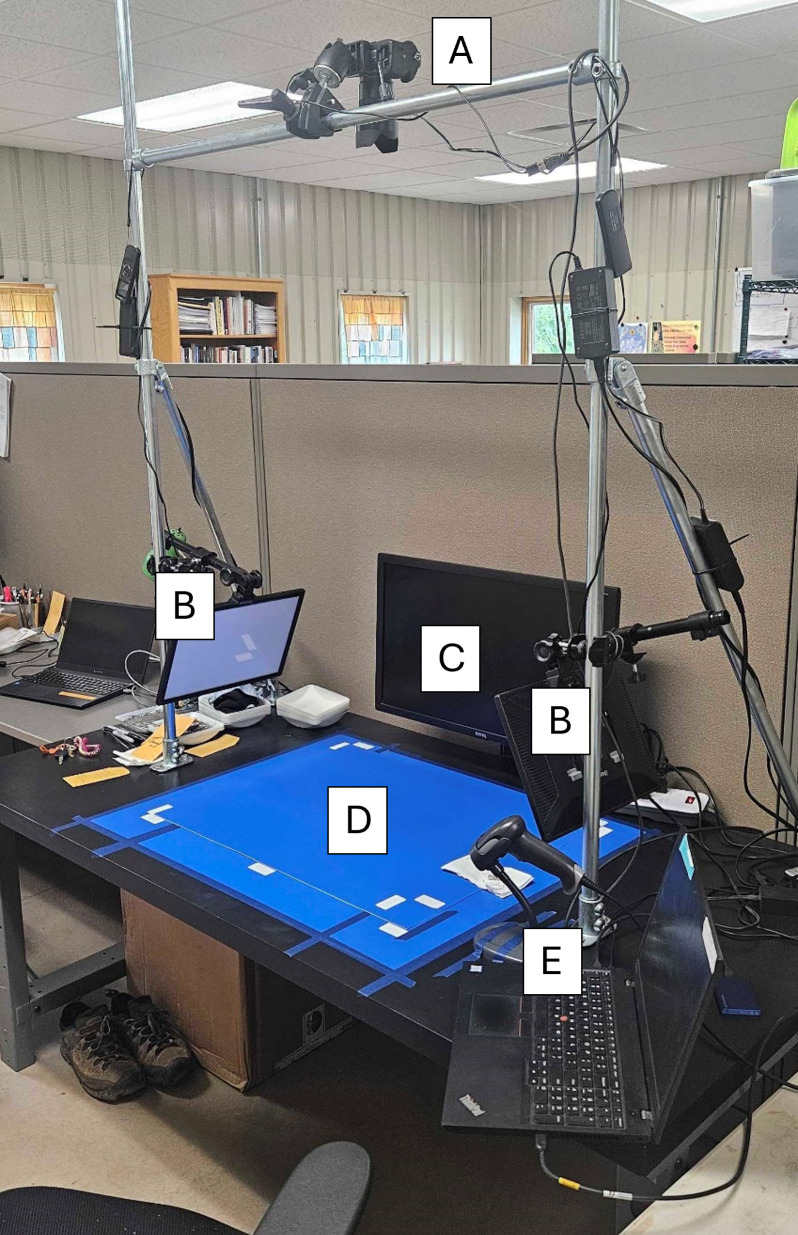
Sainfoin seed image acquisition setup with **(A)** Sony model ILCE-7RM2 DSLR camera, **(B)** LED lighting panels on both side of the imaging platform, **(C)** Camera preview monitor, **(D)** solid blue photography background mat, and **(E)** barcode scanner and laptop for running camera utilities.

The processed mixture of seeds was scattered onto a blue chroma, photography platform illuminated by two LED lighting panels from the sides (See **Figure 1**). The mixtures were imaged using a DSLR camera (Sony model ILCE-7RM2, Sony Electronics Inc., New York) with a fixed focal length 55mm lens mounted on a fixed rig directly above the platform. All images were acquired in TIFF format at ISO 100 and a 1/40s exposure time with a final resolution of 7968x5320. The images were converted to JPEG format before annotation.

### Image labeling

The images were uploaded to Labelbox, an online data annotation platform (Labelbox Inc., San Francisco, CA, USA). Bounding boxes were drawn around each seed derived object in every image and classified into one of three categories: IP (intact pods), WS (whole seeds), and SS (split seeds). IP were categorized as intact or partially intact sainfoin seed pods (IP) which still contained a single seed. WS were defined as seeds with an intact seed coat which were completely separated from the seed pod carpels. Finally, SS were defined as seeds without a seed coat that were either intact (both halves of the seed together), split in half, or fractured into small pieces. Small pieces of threshed seed pods, empty seed coats, or other pieces of seed derived material which could not be identified were left unlabeled.

We hand labeled a random selection of 20% of the images, developed a preliminary faster-RCNN model as described below, and then ran inference on the remaining 400 images to predict any seed objects, discarding any objects with a confidence score less than 0.8. We then uploaded the predictions to Labelbox using the Labelbox Python SDK to use as model assisted labels. All bounding boxes and object classifications underwent manual quality control. Adjustments, reclassifications, or removals were performed as necessary to correct inaccuracies in the preliminary model outputs and to address any errors in class assignment. Finally, all the annotations were exported into the popular COCO JSON format for use in the modeling scenarios. In total, 75,342 objects covering three classes were annotated with bounding boxes in all 500 images. The counts were based on the total number of annotated objects within each class. IP accounted for almost 48.6% of the total number of objects across all methods and varieties, while WS and SS percentages were much lower at 33.8% and 17.6%, respectively (**Table 1**).

**Table 1:** Total ground truth label counts for each class and the percentage composition within the full dataset.

Object count	Object count	Class percentage
Intact Pod (IP)	36,599	48.58%
Whole Seed (WS)	25,488	33.83%
Split Seed (SS)	13,255	17.59%
Sum	**75,342 **	**100.00%**

Values in bold are the sum total of object counts across classes and sum of individual class percentages.

### Faster RCNN modeling

The Python package scikit-learn (Pedregosa et al., 2011) was used to create training and validation image sets in an 80/20 split that were stratified equally over the ‘variety’, ‘sample-size’, and ‘processing-method’. The final training and validation dataset sizes were 400 and 100 images each, respectively. We further subsampled the training and validation sets so we could determine a minimum image set size to train an accurate model. Subsampling proportions were set at 0.05, 0.10, 0.25, 0.50, and 1.0 of the entire dataset splits (**Table 2**).

**Table 2:** The total number of images in each training and validation split for each proportion of the dataset used to train models.

Proportion	Training images	Validation images
0.05	20	5
0.10	40	10
0.20	80	20
0.50	200	50
1.00	400	100

**Figure 2:**
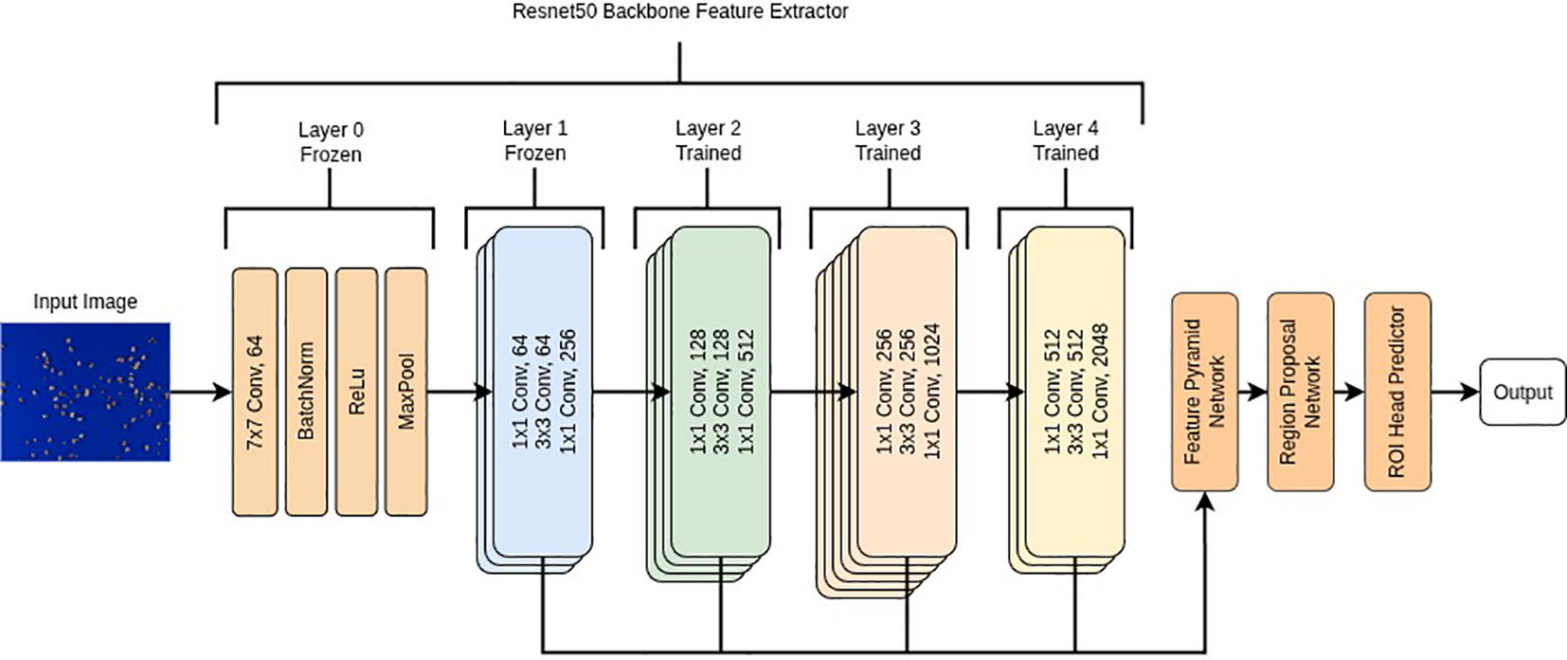
Flow diagram of the Faster R-CNN model with a ResNet50 backbone. The initial convolutional stem (Layer 0) and the first ResNet stage (Layer 1) were frozen to preserve low-level pretrained features, while Layers 2–4 were fine-tuned to adapt higher-level representations to the dataset. The outputs from Layers 2–4 were combined by a Feature Pyramid Network (FPN) to generate multi-scale feature maps, which were processed by the Region Proposal Network (RPN) and ROI head to produce final object predictions.

Object detection models were developed using a transfer learning approach within Pytorch v2.0.1 (Paszke et al., 2019) in Python 3.11.5 (Python Software Foundation, 2024). We used a torchvision Faster RCNN model (Ren et al., 2017) with an Imagenet1k v2 pretrained Resnet50 backbone (He et al., 2015), a model previously used to detect a wide variety of seed objects (Wang et al., 2022; Ouf, 2023; Islam et al., 2024). We froze all but the final 3 convolutional stages in the backbone allowing us to finetune the feature extractor and ROI head on our dataset as shown in **Figure 2**. In addition, we changed the default anchor sizes of the region proposal network from [32, 64, 128, 256, 512] to [8, 16, 32, 64, 128] to detect smaller objects in the image. Since the original image size (W 7968 x H 5320) was very large relative to the average seed object bounding box size (40 x 40 pixels), we did not resize the images before training as this would have significantly reduced the seed object sizes (e.g. when resized to 1024 x 1024, bounding boxes would be in the range of 5–7 pixels wide). All pixel values were normalized between 0–1 prior to model training.

The models were optimized using standard stochastic gradient descent (SGD) with a learning rate of 0.01 and an exponential learning rate scheduler and trained for a total of 100 epochs. We logged batch and epoch training and validation loss to Tensorboard. The model configuration was changed to return a total of 500 detections to allow for each object in the larger sample sizes to be detected. We filtered out predictions with confidence scores lower than 0.1, and then applied non-maximum suppression to the detections with an IOU threshold of 0.5 to discard overlapping predictions. Using these predictions, we calculated the mean intersection over union (mIOU), and “macro averaged” mean average precision (mAP) metrics for the validation dataset on both a global and per class basis. Preliminary experiments were run in a Google Colab environment (Google Colaboratory, 2024) with an A100 GPU to rapidly test training parameters in a Jupyter notebook setting. All final models were trained in an Ubuntu 22.04 Linux environment with an AMD Ryzen 7–7840 CPU, 64Gb of RAM, and an NVIDIA RTX 4060 GPU with single image batch size. Inference was conducted in the same environment used for final training.

**Figure 3:**
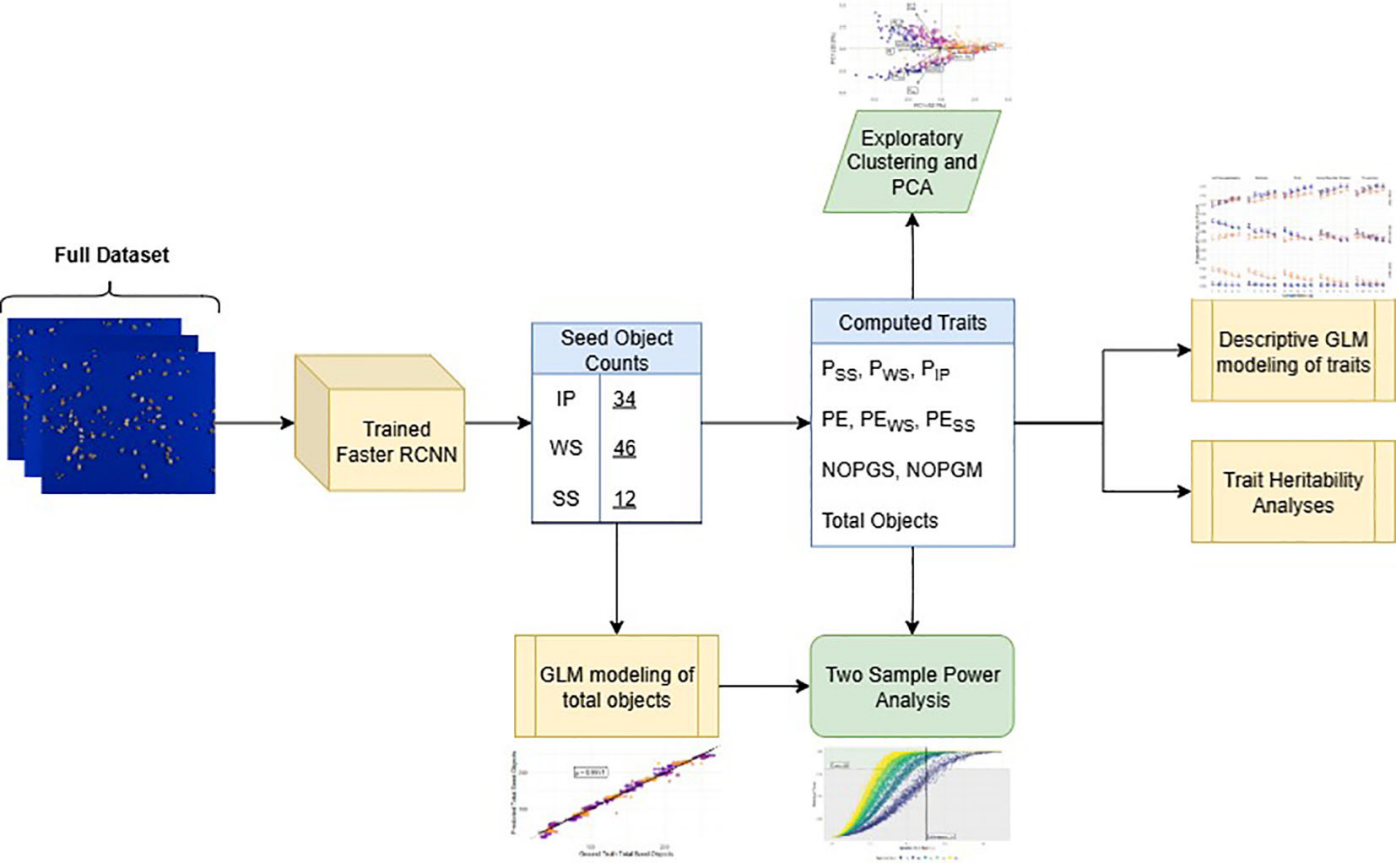
Flowchart of the analysis pipeline from images to final modeling results. The trained Faster R-CNN model produced seed object counts, which were used to compute seed traits (P_SS_, Proportion of split seeds; P_WS_, Proportion of whole seeds; P_IP_, Proportion of intact pods; PE, Processing efficiency; PE_WS_, Split seed penalized PE; PE_SS_, Whole seed penalized PE; NOPGS, Number of seed objects per gram sample; NOPGM, Number of seed objects per gram processed mixture; and Total Objects, Total number of predicted objects). These trait values informed exploratory analyses (clustering and PCA), trait GLM modeling, and heritability estimation. Total seed object counts were modeled using a GLM and combined with the computed traits for PE to conduct two-sample power analyses.

To contextualize the flow of analyses described in the sections below, **Figure 3** provides an overview of how outputs from the Faster R-CNN model were integrated with downstream methods, including trait computation, exploratory clustering, statistical modeling, heritability analyses and power analyses.

### Image inference and calculations

The final object detection model was used to run inference over the entire dataset. We output images with bounding boxes of all predicted objects, labeled by their predicted class and confidence scores. The number of detected objects in each image classified as IP or WS was recorded as-is, while the total number of objects predicted in the SS was recorded as $\left[\frac{SS}{2}\right]$ under the operating assumption that each SS object detected was one of two halves of a single WS. The total number of objects of all classes was summed (“total_objects”) and the proportions of each class in the image were calculated. This was repeated for all 500 images in the dataset. We calculated the number of objects detected per gram of fruit pod sample (NOPGS) and the number of objects detected per gram of processed mixture (NOPGM).

Dehulling efficiency (*DE*, Equation 1) can generally be defined based on the mass or on total counts of WS and SS as


(1) $$\begin{array}{c} DE=\frac{SS}{WS+SS} \end{array}$$

where SS and WS are either counts or weights of SS and WS, respectively (Wang, 2008; Hlavangwani et al., 2025). Since the preliminary processing methods we are testing result in depodding as well as dehulling seeds, we defined *PE* in Equation 2 as


(2) $$\begin{array}{c} PE=\frac{WS+SS}{IP+WS+SS} \end{array}$$

further simplified in Equation 3 as 


(3) $$\begin{array}{c} PE=P_{WS}+P_{SS} \end{array}$$

where P_WS_ and P_SS_ represent the proportions of the WS and SS in relation to the total object count in a sample. *PE* is a modification of the count-based efficiency metric in Hlavangwani (2025) which can generally express how easy it is to process legume pods. As processing needs may be different based on the desired end product, whether WS or SS, we also calculated *PE* with a penalty parameter λ ∈ [0, 1] for representing the amount of penalty to apply to the proportion of either WS or SS. Equation 4 (*PE_WS_*) shows the calculations made to penalize the proportion of SS and reward the proportion of WS in the mixture while Equation 5 (*PE_SS_*) shows the penalty applied when SS are the desired outcome of the processing method. For our experiments we chose a static value of λ = 0.6 for both *PE_WS_* and *PE_SS_*, for a more balanced penalty, but these could easily be tuned for more specific cases.


(4) $$\begin{array}{c} PE_{WS}=P_{WS}+P_{SS}(1-\lambda ) \end{array}$$

(5) $$\begin{array}{c} PE_{SS}=P_{WS}(1-\lambda )+P_{SS} \end{array}$$

### Statistical analysis

Standard generalized linear regression models (GLMs) with the structure *total_objects_i_ = β_0_ + β_1_·variety_i_ + β_2_·processing_method_i_ + β_3_·(variety_i_×processing_method_i_) + β_4_·sample_mass_i_ + ϵ_i_* were used to model the ‘total_objects’ (sum of IP, WS, and SS) and estimate the fixed effects of ‘variety’, ‘processing-method’ and their interaction ‘variety’ X ‘processing-method’, while controlling for ‘sample-mass’. While we expected a strong linear relationship between sample mass and seed object counts, we fit two models to the training data subset, one with a Gaussian (linear) link function and another with a Poisson link function, commonly used for count data. The root mean square error (RMSE) and mean absolute error (MAE) were used to compare the two models evaluated on the validation set. The model with the lowest combined metrics was chosen and retrained on the entire dataset. A two-way, type I ANOVA was used to determine significant factors.

GLMs with logit link functions were fitted to the proportional count data for IP, WS, and SS to determine the effect of ‘variety’, ‘sample-size’, and ‘processing-method’ on the outcomes and stability of these proportional estimates. Likelihood ratio tests (LRT) were used to test the models against the null fit. Model goodness of fit (GOF) was determined using the Hosmer-Lemeshow test (Hosmer et al., 2013), and Nagelkerke’s Pseudo-R2 was used to determine how much deviance the model accounted for. The estimated marginal means were used to conduct *post-hoc* analyses for means separation using pairwise multiple comparisons with a Tukey correction. *PE* estimates were also analyzed using logistic regression models as described above. All models were checked for the ANOVA assumptions before proceeding to *post-hoc* analyses.

Repeatability (R) was calculated for the *PE* estimates using linear mixed models with the ‘variety’, environment’, and ‘variety’ X ‘environment’ as random effects where environment was set as a concatenation of the ‘processing-method’ and ‘sample-size’. R was calculated for a given trait as show in Equation 6.


(6) $$\begin{array}{c} R_{GxE}=\frac{\sigma_{G}^{2}}{\sigma_{G}^{2}+\frac{\sigma_{E}^{2}}{e}+\frac{\sigma_{GxE}^{2}}{e}+\frac{\sigma_{resid.}^{2}}{re}} \end{array}$$

where $\sigma_{G}^{2}$ is the genetic variance component, $\sigma_{E}^{2}$ , is the environment variance component, $\sigma_{GxE}^{2}$ is the GxE variance component, $\sigma_{resid.}^{2}$ is the model residual variance, and *r* and *e* are the total number of individual replications and unique environments, respectively.

We performed a principal component analysis (PCA) as an exploratory visualization of multivariate trait structure to complement the formal statistical modeling of these variables. The PCA matrix contained the relativized trait values for P_IP_, P_WS_, P_SS_, *PE*, *PE_WS_*, *PE_SS_*, NOPGS, NOPGM, and the total number of seed objects for all observations. Variables were mean centered and scaled to unit variance prior to analysis. The first two principal components were extracted and plotted on the *x* and *y* axes with the rotational loadings of the variables projected onto them to form a biplot, and individual data points were colored according to the ‘variety’ and ‘processing method’. We performed a principal component analysis (PCA) to determine the main structure of the data and how the variety and processing method groups relate to the variable loadings. To assess how well the principal component space distinguished processing methods, we fit a support vector machine (SVM) model with a linear kernel to the first two principal components. Model performance was evaluated by the classification accuracy for separating data points according to processing method.

Additionally, we used the total within group sum of squares (TWGSS), commonly used as an objective function in K-means clustering, as a metric to characterize varieties using NOPGS and NOPGM, and calculated as shown in Equation 7


(7) $$\begin{array}{c} TWGSS=\sum_{i=1}^{C}\sum_{j=1,x_{j}\in C_{i}}^{N_{i}}∥x_{j}-\mu_{i}{∥}^{2} \end{array}$$

where *C* is the total number of classes, *C_i_* is the set of datapoints belonging to the *i* ^th^ class, ***x*** *_j_* is the *j* ^th^ datapoint in the class out of *N*_i_, and ***μ***_i_ is the centroid for the *i* ^th^ class.

### Power analysis

We conducted a power analysis and developed intuitive visualizations to evaluate our ability to detect differences in *PE* across varieties and methods, with respect to fruit pod sample mass. Our goal was to determine the minimum sample mass (in grams) required to confidently detect an absolute difference of 0.25 in *PE* between two samples, with a statistical power of at least 0.8. Briefly, within each sample size group (1g - 5g, n=100 per group) we calculated un-ordered absolute pairwise effect sizes resulting in a total of $\left(\frac{n}{2}\right)$ values per group. The absolute, two proportion effect size $h_{i,j}$ was calculated as shown in Equation 8


(8) $$\begin{array}{c} h_{i,j}=|\phi_{i}-\phi_{j}| \end{array}$$

where $\phi_{i}=2\arcsin(\sqrt{p_{i}})$ as described in Cohen (1988), with the constraints *i* ≠ *j*, $h_{i,j}=h_{j,i}$ for each sample pairwise calculation. The non-centrality parameter is calculated as shown in Equation 9


(9) $$\begin{array}{c} \delta =\frac{h_{i,j}}{\sqrt{\frac{1}{n_{i}}+\frac{1}{n_{j}}}} \end{array}$$

where $n_{i}$ and $n_{j}$ are the total number of objects counted in the *i* ^th^ and *j* ^th^ samples, respectively. Statistical power for each comparison was calculated using the ‘pwr.2p2n.test’ from the ‘pwr’ package as a two-sided test using the formula shown below in Equation 10


(10) $$\begin{array}{c} P_{i,j}=\Phi (\delta -z_{1-\alpha /2})+\Phi (-\delta -z_{1-\alpha /2}) \end{array}$$

Where Φ is the normal CDF, and $z_{1-\alpha /2}$ is the critical z-value for a two-tailed test.

We also simulated two-proportion theoretical power curves based on equal sample sizes from 1 to 300 total objects and then calculated the minimum proportional difference in *PE* that could be detected with 80% power at each sample size. A given effect size between two proportions near the extreme values of 0 and 1 is easier to detect compared to proportions near 0.5 due to the structure of proportional variance calculations $\left(\sigma_{p}^{2}=\frac{p(1-p)}{n}\right)$ leading to higher statistical power at the extremes of the binomial distribution. We calculated a best-case scenario when the proportional difference was centered symmetrically on 0.85 (near the extreme) and a worst-case scenario centered on 0.5, where statistical power is lowest. Finally, we plotted out these values and related them to the minimum sample size needed to detect a *PE* difference of 0.25 under both scenarios.

### Software packages

All deep learning models were trained in Python 3.11.5 with Pytorch 2 (Paszke et al., 2019), using the packages ‘OpenCV’ (Bradski, 2000), ‘Torchmetrics’ (Detlefsen et al., 2022) and ‘Scikitlearn’ (Pedregosa et al., 2011). Data cleaning, generalized linear modeling, PCA, power analyses, and graphing were conducted using R 4.4 (R Core Team, 2025) in an RStudio environment (Posit team, 2025) using a mixture of packages ‘caret’, ‘lme4’, ‘tidyverse’, and ‘pwr’ (Kuhn, 2008; Bates et al., 2015; Wickham et al., 2019; Champely et al., 2020). Schematics and diagrams were created using ‘Draw io’ (JGraph, 2021).

## Results

### Object detection modeling and inference

The training and validation loss decreased sharply for the first ten epochs, after which the loss values started to stabilize and were fully stable around 50 training epochs. The lowest training and validation losses observed were with the model trained on the entire dataset. However, the relative difference between the loss from models trained on smaller proportions of the image set, excluding the smallest subset size, were negligible.

On average, inference took 100–105 ms per image in our environment. The overall mIOU score as well as class based mIOU metrics were calculated for each of the models after training. The results are shown in (**Table 3**). mIOU was generally in the range of 0.8-0.9 and increased slightly with increasing dataset size. The highest overall mIOU (0.8926) was achieved when the model was trained on 100% of the total dataset but was not drastically higher than the overall mIOU on 50% of the data (0.8905). Class based mIOU was highest for IP (0.9046) and lowest for the SS (0.8732), which mirrors the unbalanced distribution of IP, WS, and SS annotations in the dataset. mAP scores are presented for each class, the overall average, and across objects with 50% and 75% IOU (**Table 4**). IP mAP peaked at 0.6994 at 50% of the dataset, while WS and SS had the highest mAP (0.7212 and 0.7136, respectively) at 100% of the dataset. The overall mAP macro averaged across classes was lowest when trained on 5% of the data (0.6620) and highest at 100% of the data at 0.7113. mAP50 and mAP75 both peaked at 0.9831 and 0.9257, respectively, at 50% of the data.

**Table 3:** Mean Intersection over Union (mIOU) values between the predicted bounding boxes and ground truth for each class of seed object in the dataset.

Object class	*Percentage of dataset*				
	5%	10%	20%	50%	100%
Intact Pod (IP)	0.8915	0.8962	0.9004	0.9032	0.9046
Whole Seed (WS)	0.8689	0.8751	0.8789	0.8829	0.8852
Split Seed (SS)	0.8519	0.8610	0.8639	0.8695	0.8732
Mean	**0.8772**	**0.8831**	**0.8869**	**0.8905**	**0.8926**

Columns represent the percentage of the full training and validation dataset that were used to train the model. Values in bold are the average mIOU values across classes.

**Table 4:** Mean Average Precision (mAP) per class values at 50% and 75% mIOU threshold.

Object class	*Percentage of dataset*				
	5%	10%	20%	50%	100%
Intact Pod (IP)	0.6679	0.6742	0.6908	0.6994	0.6992
Whole Seed (WS)	0.6720	0.6995	0.7050	0.7167	0.7212
Split Seed (SS)	0.6462	0.6705	0.6831	0.7059	0.7136
Mean	**0.6620**	**0.6814**	**0.6930**	**0.7073**	**0.7113**
mAP 50	0.9104	0.9039	0.9641	0.9831	0.9656
mAP 75	0.8269	0.7936	0.8915	0.9257	0.9103

Columns represent the percentage of the full training and validation dataset that were used to train the model. Values in bold are the overall mAP values 'macro averaged' across all classes.

**Figure 4:**
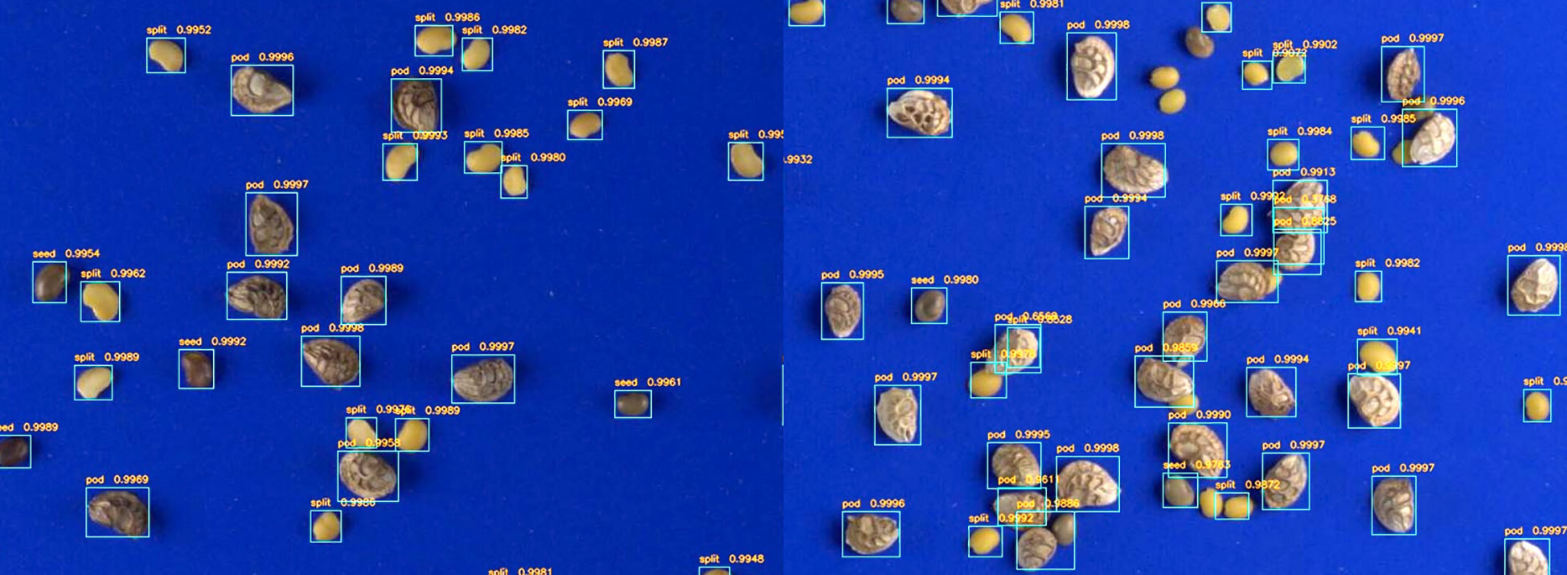
Faster RCNN predictions on representative images from left to right with no crowding **(A)** and some crowding **(B)**. In situations with crowding, the minor class is sometimes misclassified or remains undetected.

Both mIOU and mAP were highest in the models trained on 50% and 100% of the data. We selected the model trained on the full training set as the final model to use for inference for all downstream analyses. After processing all the images through the model, we used the resulting predictions to draw bounding boxes over all images. Almost every seed object was detected properly and with reasonable bounding boxes (**Figure 4A**). In a few cases the model had a difficult time identifying SS, particularly when they were touching or when there were tightly clustered groups of objects as in **Figure 4B**.

### Predicted seed object counts

**Figure 5:**
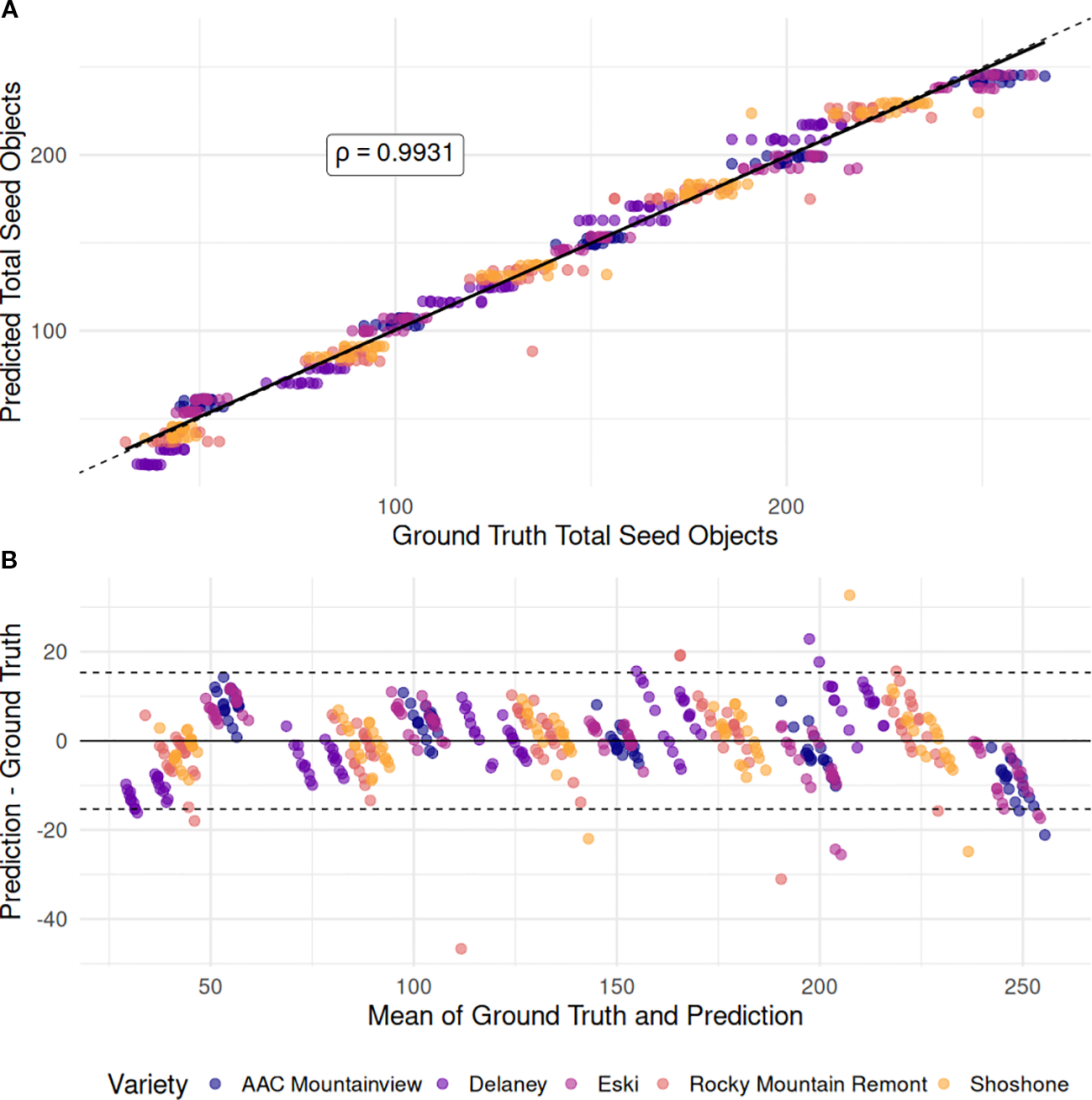
Comparison of total object counts with GLM-predicted seed object counts. **(A)** Scatterplot showing the linear relationship between the ground-truth total seed objects detected in each image (x-axis) and the seed object counts predicted by the GLM model (y-axis), which included ‘variety’, ‘processing method’, and ‘sample mass’ as predictors. The solid black line represents the fitted regression line between the two variables, while the dotted line indicates the 1:1 correspondence line (perfect agreement). **(B)** Bland–Altman plot assessing agreement between ground-truth and predicted counts. The x-axis shows the mean of each ground-truth–prediction pair, and the y-axis shows the difference between prediction and ground truth. The dotted lines indicate the limits of agreement, defined as ±1.96 × SD of the differences.

The total object counts from the FasterRCNN model output $\left(IP+WS+\frac{SS}{2}\right)$ was modeled using generalized linear models with factors ‘variety’, ‘processing-method’ and their interaction plus a controlling term for ‘sample-mass’ with both linear and Poisson link functions. While Poisson regression is standard for modeling counts and our model fit had a high pseudo-R^2^ value (0.948), the model predictions for the holdout set were not sufficiently accurate (RMSE = 14.467, MAE = 13.085), and it failed to outperform the standard linear model (R^2^ = 0.986, RMSE = 6.874, MAE = 5.316). **Figure 5** shows the highly linear relationship between the total object count and the GLM model predictions (ρ=0.9931) and the Bland-Altman measurement correspondence between the ground truth and GLM predictions. After refitting the linear GLM on the entire dataset, an ANOVA revealed that the ‘variety’ was highly significant (*p* < 2.2e-16), as was the ‘processing-method’ (*p* = 4.42e−15), when controlling for ‘sample-mass’. There was no evidence of their interaction (*p* = 0.238). The mean seed object counts for each sample size, marginalized over method, and variety were 46, 92, 137, 183, and 230 for 1g - 5g, in order – on average an increase of 46.12 seed objects in the processed mixture. However, this varied drastically across varieties. ‘Eski’ and ‘AAC Mountainview’ had the highest seed object counts per gram increase in fruit pod sample mass (60 total detected objects) compared to varieties like ‘Delaney’ and ‘Rocky Mountain Remont’ (32 and 42 total detected objects) when keeping other factors constant. Interestingly, processing the same pod sample mass by different methods resulted in differing amounts of product loss. Impact dehulling resulted in ≈ 4 fewer detected objects compared to belt threshing - a small effect size, but highly significant (*p* = 4.42e−15).

### Seed object analysis

We hypothesized that the proportional estimates of each class of objects in the processed mixture would stabilize with increasing sample size, i.e. a given estimated mean would converge to one reliable value per ‘variety’ X ‘processing-method’ combination and would have lower variance in the estimates across the 10 replicates. We performed some descriptive statistical analysis of this experiment to validate this. Three GLM models were fit to the P_IP_, P_WS_, P_SS_ data were - all well specified (LRT *p*-values « 0.0001, Hosmer-Lemeshow *p*-values ≈ 1, Nagelkerke pseudo-R^2^ ≥ 0.99). However, in general, the value of seed object proportional estimates did not stabilize as sample size was increased but tended to either increase or decrease linearly with changes in sample mass as described below.

**Figure 6:**
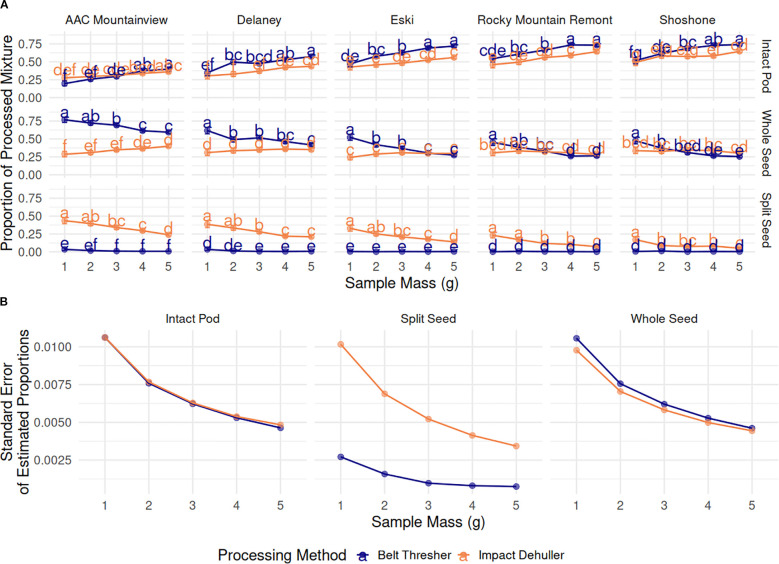
**(A)** Estimated marginal means (proportion) ± 95% CI of the proportions of processed mixture objects (grid rows), by sainfoin variety (grid columns), processing method (blue=belt thresher, green=impact dehuller), and sample mass (g) on the x-axis. Means within a subplot that have letters in common were not shown to be statistically significantly different from each other at α=0.05. **(B)** Standard error of the estimated marginal means of the proportions of processed mixture objects compared across sample mass (g) and marginalized over varieties.

The proportion of IP in the resulting processed seed mixtures increased across all varieties, regardless of the processing method as sample mass increased (**Figure 6A**). The variety ‘AAC Mountainview’ had the lowest proportion of IP in the mixture after processing (0.308) while varieties ‘Rocky Mountain Remont’ and ‘Shoshone’ had the highest (0.607 and 0.621, respectively) marginalized across processing method and sample mass. The processing method also significantly impacted the IP proportions - the belt thresher tended to leave more IP intact when compared to the impact thresher (0.546 versus 0.459, *p* < 2.2e-16). Within each variety, belt threshing always resulted in higher IP counts compared to the impact dehuller, except in the case of ‘AAC Mountainview’, where the estimates for each method were similar (see **Figure 6A**). **Table 5** shows the marginalized means across sample sizes. Processing by belt thresher resulted in a wider range in the IP proportions between varieties from 0.299 (‘AAC Mountainview’) to 0.662 (‘Shoshone’), whereas the impact dehulling proportion range was more constrained from 0.316-0.577.

**Table 5:** Estimated marginal means of seed object proportions marginalized over sample mass.

Variety	Method	Intact pod	Whole seed	Split seed
AAC Mountainview	Belt Thresher	0.299 ± 0.013 d	0.682 ± 0.013 a	0.015 ± 0.003 a
Delaney	Belt Thresher	0.484 ± 0.015 c	0.501 ± 0.015 b	0.012 ± 0.003 a
Eski	Belt Thresher	0.622 ± 0.013 b	0.373 ± 0.013 c	0.005 ± 0.002 b
Rocky Mountain Remont	Belt Thresher	0.661 ± 0.013 a	0.335 ± 0.013 d	0.004 ± 0.002 b
Shoshone	Belt Thresher	0.662 ± 0.013 a	0.331 ± 0.013 d	0.006 ± 0.002 b
AAC Mountainview	Impact Dehuller	0.316 ± 0.013 d	0.341 ± 0.013 a	0.338 ± 0.012 a
Delaney	Impact Dehuller	0.371 ± 0.015 c	0.340 ± 0.015 a	0.282 ± 0.013 b
Eski	Impact Dehuller	0.491 ± 0.014 b	0.287 ± 0.012 c	0.215 ± 0.011 c
Rocky Mountain Remont	Impact Dehuller	0.550 ± 0.014 a	0.310 ± 0.013 bc	0.132 ± 0.009 d
Shoshone	Impact Dehuller	0.577 ± 0.014 a	0.329 ± 0.013 ab	0.088 ± 0.008 e

Values presented are the marginal means ± 95% CI of the estimate. Means within a processing method that share a common letter were shown not to be statistically significantly different from each other at α=0.05.

The overall trend in change for whole seed proportions depended on the method. In belt threshed samples, whole seeds decreased in number with increasing sample size across all varieties - 0.573, 0.480, 0.441, 0.374, and 0.353 for 1-5g, respectively, marginalized across varieties. However, proportions stayed mostly constant for impact threshed samples with values ranging from 0.296 to 0.334 for all varieties and sample sizes, and there were very few significant differences across sample sizes within each variety (**Figure 6A**). On average, belt threshing resulted in a larger proportion of whole seeds (0.443) compared to impact dehulling (0.321, *p <*2.2e-16). ‘Rocky Mountain Remont’ and ‘Shoshone’ had the lowest proportions of whole seeds (0.322 and 0.330) compared to ‘AAC Mountainview’ and ‘Delaney’ (means of 0.419 and 0.512). However, there was a large difference between the average seed proportion between belt threshing and impact dehulling for ‘AAC Mountainview’ and ‘Delaney’ (proportional difference of 0.341 and 0.161) that was not present for other varieties ( $\hat{\mu }$ = 0.037).

The proportion of SS in the processed mixtures was very low overall (estimated marginal mean = 0.0399, **Figure 6A**). Within belt threshed samples, SS were present in extremely low proportions relative to other seed objects (0.00715), whereas impact dehulled samples were much more likely to contain splits (0.194, marginalized across variety and sample mass). Within impact dehulled samples, varieties ‘AAC Mountainview’ and ‘Delaney’ had SS proportions of 0.338 and 0.282, estimated over all sample sizes, whereas ‘Shoshone’ contained very few SS (0.088, **Table 5**). Within belt threshed samples, ‘ACC Mountainview’ and ‘Delaney’ had the highest SS proportions ranging from 0.012-0.015, and were significantly different from the other varieties, which ranged from 0.004-0.006. Increasing the sample size had a negative impact on the SS proportions when the samples were processed by impact dehulling and decreased the average estimate from 0.301 (1g) to 0.125 (5g). Even though the proportional point estimates for each object class were not stable as the sample size was increased, the standard error of the model estimates was reduced as expected (**Figure 6B**).

### Processing efficiency calculations

**Figure 7:**
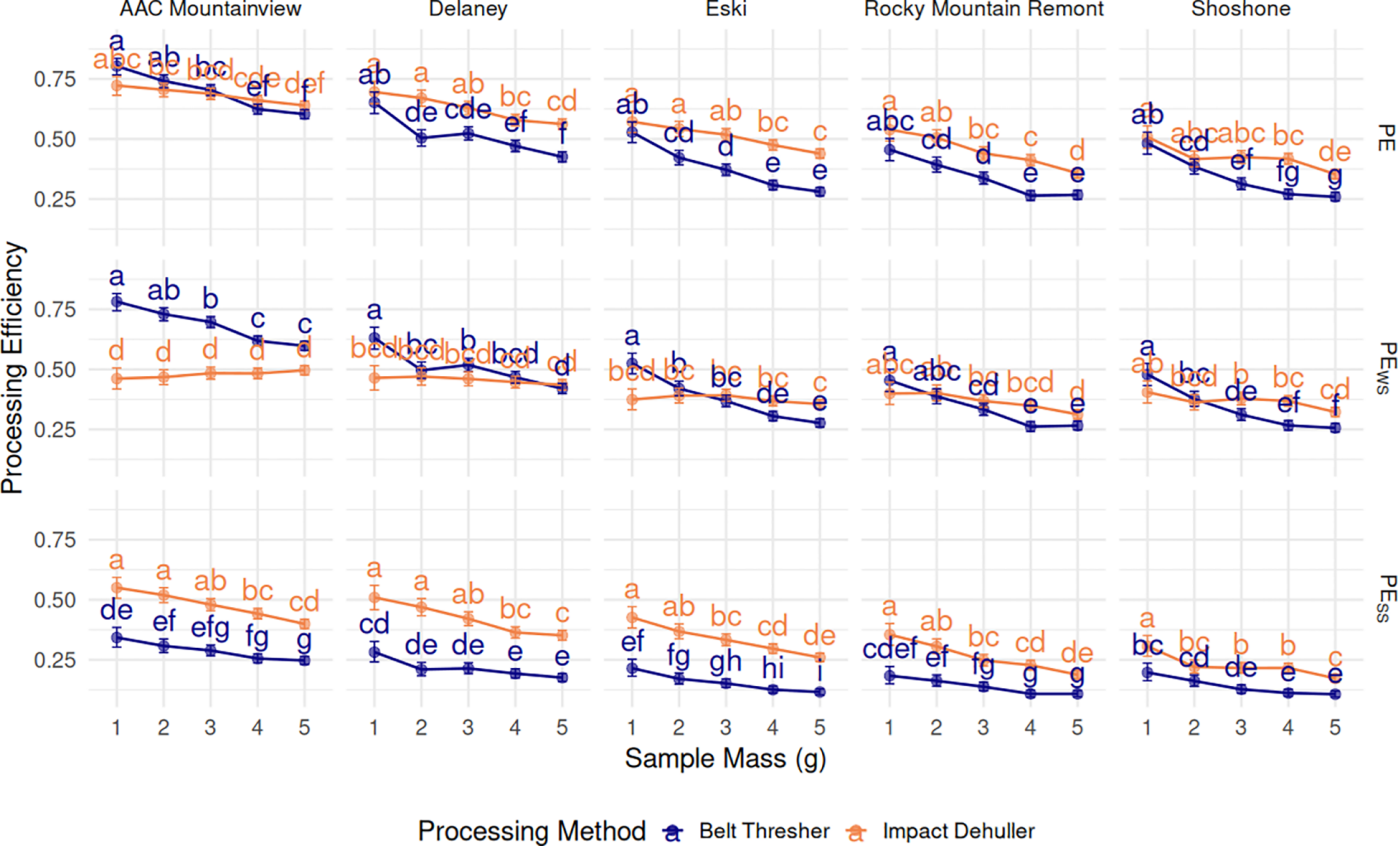
Estimated marginal means (proportion) ± 95% CI for processing efficiency metrics PE, PE_WS_, and PW_SS_, (grid rows), by sainfoin variety (grid columns), processing method, and sample mass (g) on the x-axis. Means within a subplot that have letters in common were not shown to be statistically significantly different from each other at α= 0.05.

Processing efficiency (*PE*) was calculated as the sum of the WS and SS proportion. *PE* variations *PE_SS_* and *PE_WS_* penalize higher proportions of WS and SS, respectively. Unpenalized *PE* had a negative relationship with sample mass across the methods and varieties (**Figure 7**). The marginal mean *PE* for 1g samples was 0.602 and dropped to 0.413 for 5g samples across varieties and methods. ‘AAC Mountainview’ had the highest overall *PE* at 0.692, ‘Rocky Mountain Remont’ and ‘Shoshone’ had far lower *PE* on average (0.393 and 0.379). Processing method had a small but significant effect on *PE* (*p* < 2.2e−16) - impact dehulled samples had a *PE* of 0.541 on average compared to 0.454 for belt threshed samples.

*PE_WS_* was slightly lower on average than unpenalized *PE* (0.427 versus 0.498). ‘AAC Mountainview’ still had the highest *PE_WS_* (0.588) while ‘Shoshone’ was the lowest (0.350). *PE_WS_* was had a negative correlation with sample size for belt threshed samples however, but the relationship was not as pronounced with impact dehulled samples (**Figure 7**). Marginal estimates for *PE_WS_* across varieties and sample sizes were significantly higher (*p* = 2.5e−05) for belt threshed samples (0.447) compared to impact dehulling (0.408), though the effect size was small.

The overall marginal mean of *PE_SS_* was lower than any other *PE* computations at 0.25. There were strong varietal differences, again with ‘AAC Mountainview’ having the highest estimate at 0.378 and ‘Shoshone’ the lowest at 0.176 (*p* < 2.2e−16). Belt threshed samples had significantly lower *PE_SS_* compared to impact dehulled samples (0.179 compared to 0.337, *p* < 2.2e−16) when marginalized across ‘variety’ and ‘sample-mass’. All *PE_SS_* estimates decreased with increasing sample size across varieties and methods (0.326 for 1g, 0.197 for 5g). Repeatability ( R ) was calculated for all the *PE* traits. $R_{PE}$ was close to 1 (0.9913), as was $R_{PE_{SS}}$ (0.9787). Repeatability for *PE_WS_* was still high, but slightly lower than the other two repeatability estimates (0.8702).

### Clustering and PCA

**Figure 8:**
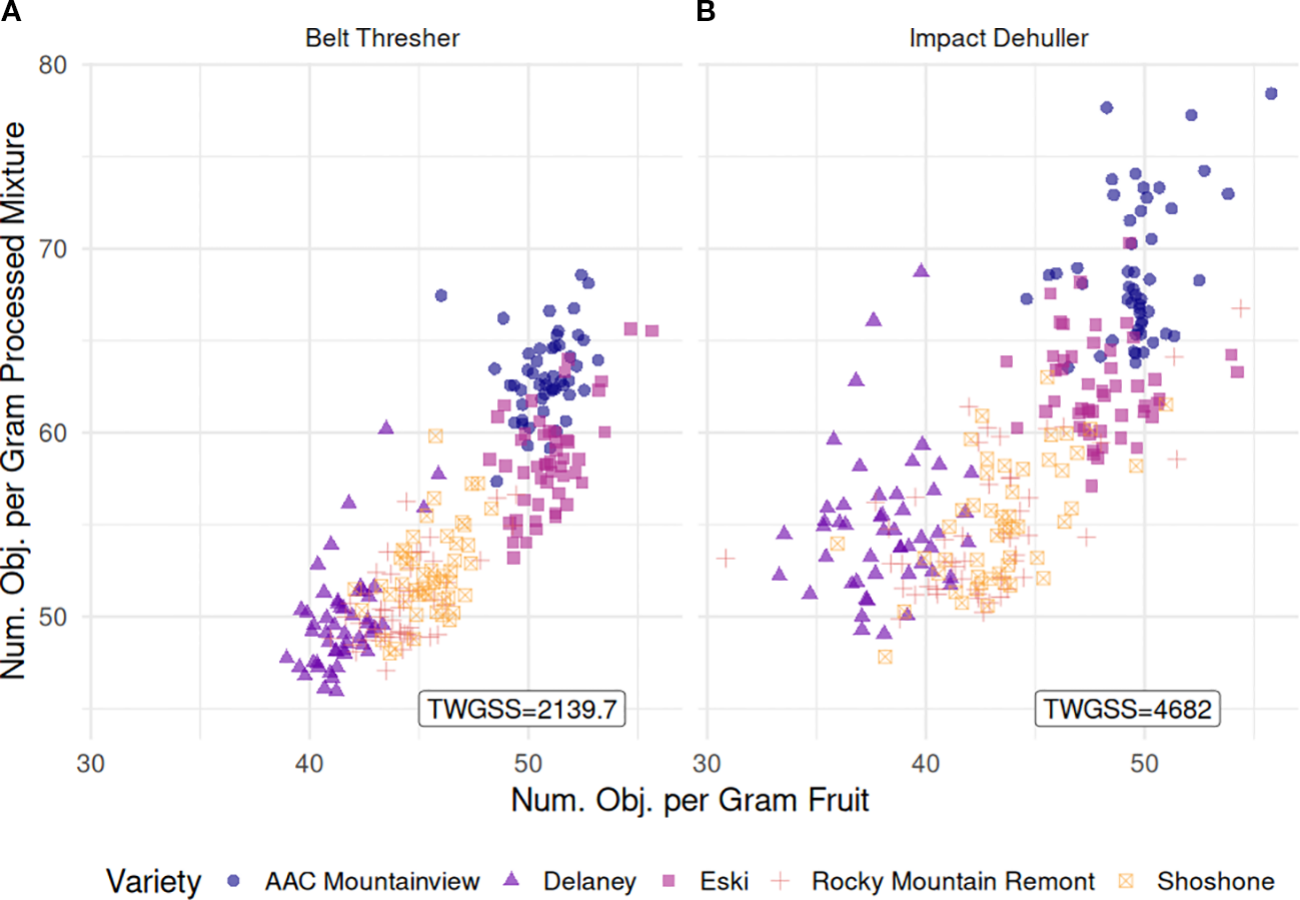
Scatterplot relating the number of objects per gram sample (NOPGS, x-axis) and the number of objects per gram of processed mixture (NOPGM, y-axis) for **(A)** belt thresher processed samples and **(B)** impact dehuller processed samples. The total number of objects is the sum of all sainfoin seed pod derived material which includes IP, WS, and SS. TWGSS is the total within variety group sum of squares used as a measure of group compactness.

**Figure 8** illustrates the relationship between the number of objects per gram sample (NOPGS) and per gram processed mixture (NOPGM) in both methods. We observed overall mean counts of 45.7 for NOPGS while NOPGM was slightly higher at 57.2. Samples processed with the impact dehuller had a lower average NOPGS (44.5), but higher average NOPGM (59.3) compared to those processed with the belt thresher (46.8 and 55.1, respectively, *p* < 2.2e-16). Varietal differences were consistent across processing methods. ‘Delaney’ exhibited the lowest counts for both NOPGS and NOPGM (39.9 and 52.4, respectively), while ‘Eski’ and ‘AAC Mountainview’ were the highest for both measurements (NOPGS = 49.5 and 50.2, and NOPGM = 60.5 and 66.0). We calculated the total within group sum of squares (TWGSS) for each processing method as a measurement of dispersion. TWGSS was substantially lower for belt-threshed samples (2139.7) than for impact-dehulled samples (4682).

**Figure 9:**
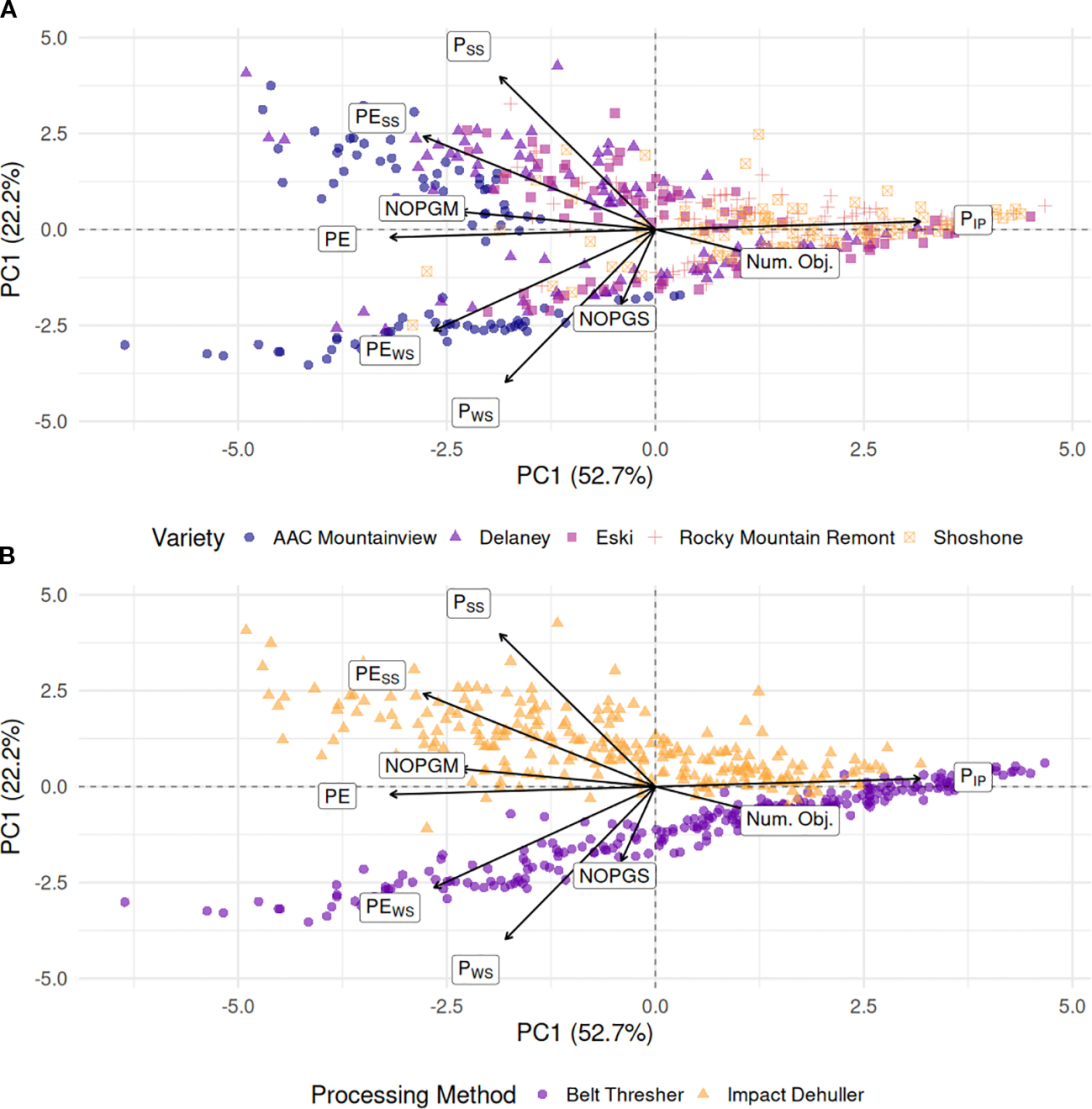
Principal component analysis (PCA) biplots of the first two components from the relative processing traits conditioned on **(A)** sainfoin variety **(B)** processing method. Arrows with labels represent the PCA loadings of each of the variables to the principal directions with longer arrows contributing more to the first two components than shorter arrows. The first two components captured almost 75% of the variance in the data P_SS_, Proportion of split seeds; P_WS_, Proportion of whole seeds; P_IP_, Proportion of intact pods; PE, Processing efficiency; PE_WS_, Split seed penalized PE; PE_SS_, Whole seed penalized PE; NOPGS, Number of seed objects per gram sample; NOPGM, Number of seed objects per gram processed mixture; Num. Obj., Total number of seed objects.

The first two PCA components capture approximately 75% of the variation in the data, and when the varieties are projected onto the components, they cluster together along the first component axis for most data points (**Figure 9A**). As revealed by logistic regression modeling, the *PE* traits contributions are strongly associated with the majority of the ‘AAC Mountainview’, ‘Delaney’, and ‘Eski’ datapoints, the three varieties with the highest *PE*. Most of the P_IP_ contribution to the data lies along PC1 and associated with varieties ‘Rocky Mountain Remont’ and ‘Shoshone’, both of which had the lowest overall *PE*. However, what is most interesting is the stark contrast in clustering of processing methods shown in **Figure 9B**. A simple linear kernel SVM trained on the first two principal components of the training set achieved 99% accuracy on the validation holdout. P_IP_ was strongly associated with most of the belt threshing datapoints and varieties with low *PE*. Whereas P_WS_ and *PE_WS_* were much more associated with belt threshed samples of ‘AAC Mountainview’ and ‘Delaney’. As expected, the main contributions of *PE_SS_* and P_SS_ lie along impact dehulled samples and away from belt threshed samples.

### Power analysis

**Figure 10:**
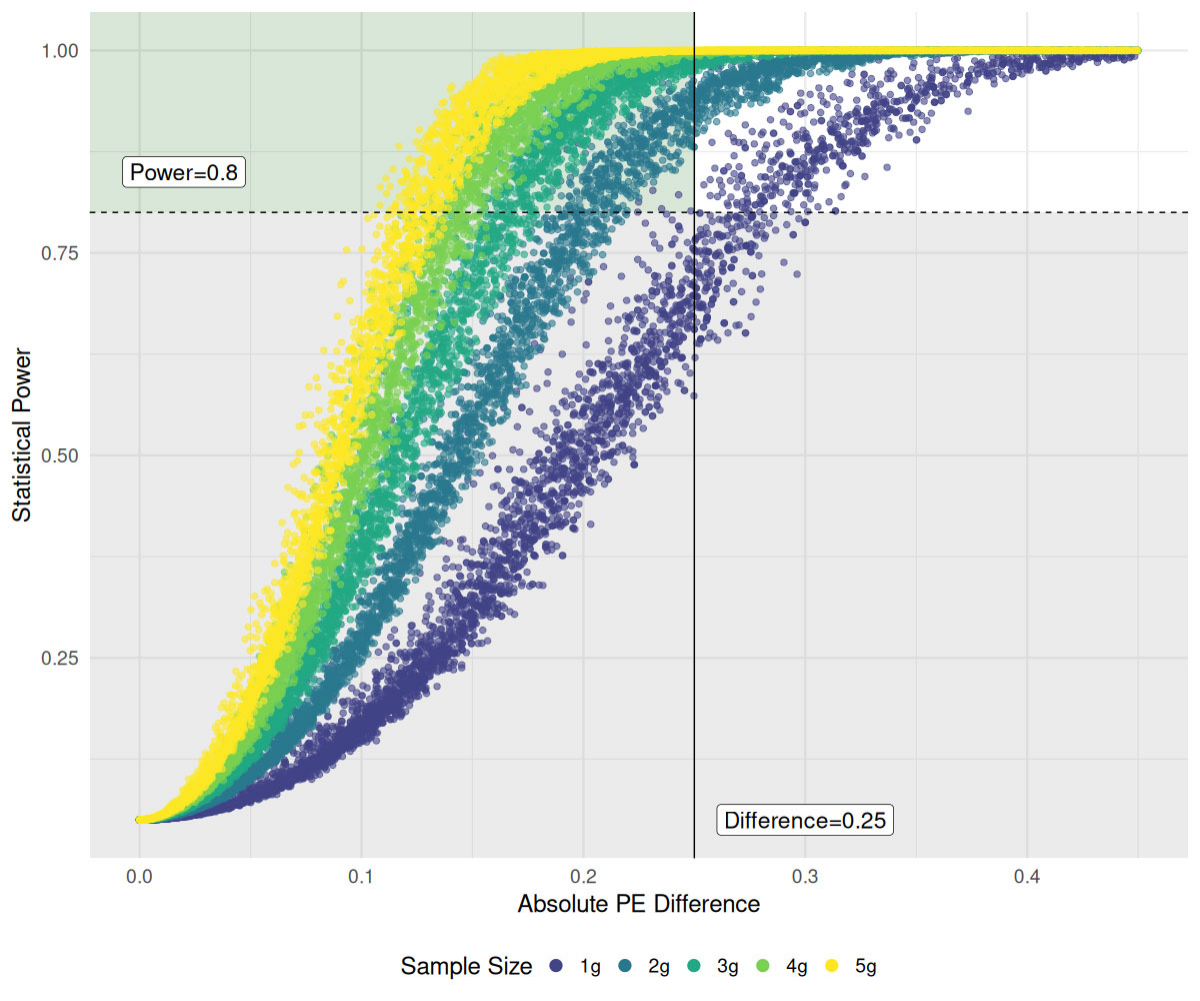
Effects of sainfoin fruit pod sample mass (g) on statistical power to detect absolute differences in PE between two samples, $\left|PE_{i}-PE_{j}\right|$ . Pairwise power tests were performed within each sample mass group. The horizontal dashed line indicates a power threshold of 0.8. The solid vertical line marks our target absolute proportional difference of 0.25 in samples’ PE. The target detection region (light green background) indicates the area where $|PE_{i}-PE_{j}|\leq 0.25$ , and $P_{i,j}\geq 0.8$ . Colors denote different sample masses. X-axis is truncated to 0.45 for clarity, at the expense of removing comparisons with large effect sizes and power nearing 1.0.

**Figure 10** shows the effect of increasing fruit pod sample size (g) on the ability to reliably detect small differences between two sample proportions of each sample’s *PE*. The greyed-out region on the lower half of the graph represents all the pairwise power tests conducted which had a power of less than 0.8. The target region, outlined in green, indicates comparisons with at least 80% statistical power and a *PE* difference no greater than 0.25. Over all sample sizes, 41.7% of comparisons made had at least 80% power, but only 17.7% of comparisons within the 1g sample size group had the same power. When only 1 gram of fruit pods was sampled and processed, only medium to larger differences in *PE* (> 0.28), on average, could be detected reliably at or above $P_{i,j}=$ 0.8 between any two processed samples *i* and *j*. Out of the 4950 $\left(\frac{n}{2}\right)$ comparisons made in the 1-gram sample group, only 17 lay in the target zone. However, as the sample size was increased to 2g, the lower limit of detection measured was a mean *PE* difference of 0.203 with 511 comparisons in the target zone, followed by 0.165 for 3g samples, 0.135 for 4g, and 0.125 for 5g. The lower detection limit gains made by increasing the sample size show a non-linear trend, with the greatest gain made when the sample size increased from 1 to 2 grams.

**Figure 11:**
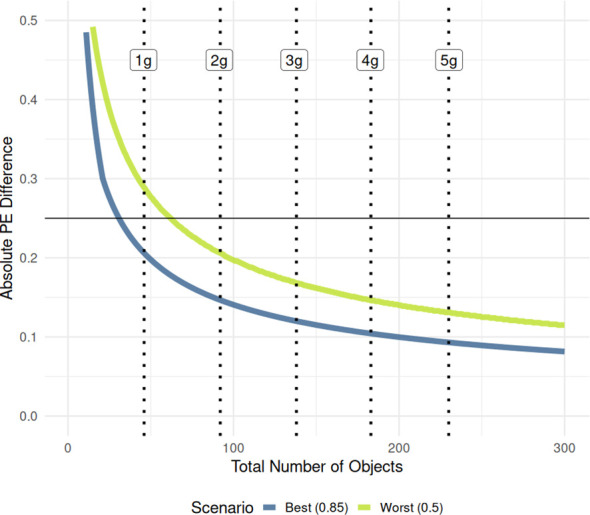
Two-proportion power curves generated for two proportional PE values with equal number of observations. Each curve’s values represent the minimum proportional difference in PE that can be detected with 80% power at the given number of observations (total number of objects in the processed mixture). Y-axis is truncated to 0.5 to only show PE differences in the intended range. Vertical dotted lines indicate the average number of total objects in the processed mixture for each sample size, marginalized across varieties.

We also simulated the power between two samples containing an equal number of detected objects, from 1 to 300, and then calculated the minimum *PE* spread detectable under two scenarios: a best-case scenario centered symmetrically at 0.85, and a worst-case scenario centered at 0.5 where proportional variance is highest and power is lowest. **Figure 11** shows the results and how they relate to sample mass. The greatest gain in detection power between two samples comes within the first gram of fruit pods sampled. While one gram of sample contains approximately 46 total objects when processed, proportional differences lower than 0.25 are only reliably detected with enough power when sample proportions are centered at the extremes (i.e. at 1 gram $|PE_{1}-PE_{2}|=0.206$ ), a relatively large effect size ( $h_{i,j}=0.617$ ). When the difference is centered on 0.5, the lower detection limit is considerably higher (at 1 gram $|PE_{1}-PE_{2}|=0.289\geq 0.25$ ) even though this corresponds to a slightly smaller absolute effect size ( $h_{i,j}=0.586$ ). However, sample sizes of at least two grams are sampled (average of 92 seed objects in the processed mixture), both the worst case and best-case scenarios are firmly below the 0.25 target difference - $|PE_{1}-PE_{2}|=0.147$ when centered at 0.85, and $|PE_{1}-PE_{2}|=0.206$ for two samples centered at 0.5. Taken together, these two curves form the range of a reasonable lower limit of detection at 80% power that can be used to find the minimum sample size needed to detect a given absolute difference in *PE* between two samples.

## Discussion

In this study, we combined deep learning–based object detection with classical statistical modeling to develop a framework for quantifying sainfoin seed pod *PE*. Our approach demonstrated that accurate seed object detection can be achieved with relatively few training images when imaging conditions are controlled, and that model-assisted labeling substantially reduces annotation burden. Through factorial experiments, we revealed that *PE* is strongly influenced by both processing method and variety, highlighting physical constraints in pod dehulling and threshing. By introducing a generalized *PE* metric, we quantified varietal differences with high repeatability and established minimum sample sizes required for robust comparisons between breeding lines. Collectively, these findings illustrate how integrating computer vision with power-based statistical principles can provide plant breeders with reliable, reproducible, and high-throughput methods for evaluating seed processing traits.

### Dataset creation

Scientific imagery has advantages over images collected “in the wild”, one being that images can be collected in as precise a format as time and budget constraints allows. Image sets collected with the same camera, software and ambient conditions allow one to train an accurate model with very few images - 80 in the present case. We recognize that images collected over a wide range of scenes, backgrounds, scales, and ambient conditions aid in training more generalizable models. But for most research settings, this may not be strictly necessary if the data acquisition pipeline is not prone to change and/or there is no evidence of model overfitting. That notwithstanding, a robust set of image augmentations as employed in the current work may be used to help regularize the model - see Kaur, Khehra, and Mavi (Kaur et al., 2021) for a review of appropriate methods. Additionally, researchers may take advantage of semi-supervised learning methods when unlabeled data far outweighs the labeled images (Sohn et al., 2020; Zhang et al., 2021). We have published our full dataset on Zenodo for further research along these lines (Meyering et al., 2023).

### Deep learning modeling

Deep learning object detection models can remove most of the dataset specific parameter tuning by learning the general features of a class of objects regardless of inconsistencies in background and other ambient conditions. However, this results in large amounts of frontloaded work for researchers due to time associated with image labeling, which could be a heavy lift for small breeding programs. Hand-labeling one image took approximately 8–9 minutes to annotate on average across sample sizes, with images from the smaller sample sizes taking considerably less time and larger sample images taking upwards of 15 minutes. To overcome this, we labeled approximately 100 images and then trained a foundation model to use for model assisted labeling. This model, while not perfect, did a fair job of pre-labeling the rest of the images. We estimated that this reduced labeling time to ≈ 1 minute per image.

Our model training experiment on 5 and 10% of the dataset resulted in models with decent performance regarding mIOU (0.877-0.883), mAP50 (0.904-0.91) and mAP75 (0.7936-0.827) (**Tables 3**, **4**). When trained on 20-100% of the dataset, the model performance improved, particularly in terms of SS detections, though models trained on the entire training set were not significantly more accurate than models trained on 50% of the training set. Since our dataset includes images taken of three standard classes against a consistent background with consistent lighting, the intra-dataset variability of the images is low, which explains the low number of images required. The training dataset size requirements would be different for images captured under more variable conditions (e.g. multiple backgrounds and image scales). Additionally, alternative classification loss functions designed to mitigate class imbalance such as recall loss or focal loss could help improve detections of the minority class, SS (Lin et al., 2017; Tian et al., 2022).

While Faster R-CNN remains a highly accurate detector, it is generally less computationally efficient than newer single-stage architectures such as EfficientDet or models in the YOLO (You Only Look Once) family (Tan et al., 2020; Jiang et al., 2022). Building on the results presented here, our dataset offers a strong basis for a systematic comparison of these architectures for small object detection in controlled imaging environments. Future work could use the current dataset to quantify performance differences in terms of accuracy, inference time, and computational requirements, clarifying the trade-offs between speed and precision. Such analyses would be particularly valuable for guiding deployment in real-time seed counting applications, including integration with seed sorters or conveyor-based sampling systems, but lay outside the scope of the present work.

### Seed object modeling

We developed a multiple regression model to predict the number of total seed objects of a given variety and sample mass so that we could use the predictions to generate power curves. Due to the linear response with sample mass, the linear model outperformed the Poisson count model, which was not surprising (**Figure 5**). The GLMs fit to the proportional data, however, were surprising. Our initial hypothesis was that at lower sample masses (1-2g) the variance of the proportional measurements over the 10 random replicates would be high but would decrease as the sample mass increased to 4-5g while the proportional estimates stayed constant. However, contrary to our hypothesis, the proportional estimates were not stable across sample sizes, though the standard error of the marginal means of the measurements did decrease as sample size increased as expected (**Figure 6**). This indicates that there are processing method-based effects on the seed proportions that are dependent on the number of pods physically fed into the instrument at one time. The clear indications of this are the increased rate of intact pods left in the dehulled mixture as the sample mass was increased. This was true across both methods and varieties. There was a strong interaction between the sample mass and method for the resulting proportion of seeds - increasing sample amount resulted in far fewer whole seeds for impact dehulling but had relatively little impact on the seed proportion estimates of all varieties for belt thresher processed samples. This is contrasted with sharp decline of split seeds for impact dehulled samples with increasing sample mass, while split seeds in the belt threshed samples were consistently low across all varieties and sample masses.

We can draw several conclusions from this. First, there are physical limitations to how well sainfoin pod samples are processed depending on the sample mass and variety. Across the board, higher proportions of IP were left in the mixture as sample mass increased suggesting either that the machines were both overloaded with sample and could not process them effectively, or that the samples were not processed for a long enough period relative to the sample mass. We set the physical parameters of the belt thresher and impact dehuller ahead of time based on running test samples of 1-2g through the instruments, as well as experience from processing 1g samples. It is highly possible that the resultant *PE* estimates would have been higher had we processed samples for a longer period. Several other studies have focused on optimizing small sample processing methods for small grains (Doehlert and McMullen, 2001; Doehlert and Wiessenborn, 2007; Oomah et al., 2010; de Figueiredo et al., 2013). However, most have focused on tuning machine parameters (timing, grain parameters, etc.) for a fixed sample size. It is reasonable to assume that sample mass specific settings would allow us to optimize our methods further. Unfortunately, we did not have the resources to investigate different machine parameters any further at the time of this project.

Second, the increase in pod proportion is offset by a decrease in the number WS for belt threshed samples and a decrease in SS for impact dehulled samples. SS were almost nonexistent in belt threshed samples, while they were much more prevalent in impact dehulled samples (**Table 5**). The WS proportion was constant and less than 0.4 in impact threshed samples. This highlights the importance of choosing the correct instrument for processing depending on the desired end product, whether WS or SS.

### Measuring processing efficiency

In contrast with other processing studies, we did not measure *DE*, but instead a new, general metric, *PE*, which includes both WS and SS in the calculation. We found that *PE* was highly dependent on the sainfoin variety and sample size (**Figure 7**). Others have observed differences in legume grain *DE* based on variety and grain quality parameters using techniques such as response surface methodology (Wang, 2005; Goyal et al., 2008; Wang, 2008; Ndukwu et al., 2019). While we did not test the grain moisture content as this was not a factor for our experimental design, all seeds were stored under the same environmental conditions for a considerable time before processing, making any measurable differences in grain moisture between varieties an unlikely cause of the observed contrasts in *PE*. Our repeatability estimates for the *PE* traits were very high (» 0.85) and indicate that while our varietal *PE* themselves were not as high as most *DE* estimates in the literature, there is a great deal of genetic variance represented in the commercial varieties tested that could be exploited in a breeding program focused on sainfoin lines with good seed processing traits.

We calculated the number of objects per gram fruit pods sample (*NOPGS*) as well as the number of seed objects per gram of processed mixture (*NOPGM*) and compared them using clustering metrics. For both methods, the relationship between them was linear, as expected, and the varieties tended to cluster together. However, the within group cluster sum of squares for the belt thresher was less than half of that for impact dehulled varieties, indicating more compact and uniform varietal processing responses under belt threshing compared to impact dehulling (**Figure 8**). This could suggest that impact dehulling creates more fractured SS (i.e. more than 2 SS per WS) and may also result in many of these SS being removed with the processing debris such as empty seed pods and seed hulls. Impact dehulling, which uses centrifugal force and impact paddles is, in general, the more forceful processing method (Singh et al., 2024; Singh and Rao, 2025) compared to belt threshing, which is focused on gently removing the outer pericarps or husks while minimizing damage to the seed (Mesquita and Hanna, 1993; Adanu et al., 2025).

We chose to use fixed equipment settings and processing times for the belt thresher and the impact dehuller based on our previous experiences processing sainfoin pods. Increasing the number of sample passes through the belt thresher could feasibly result in fewer IP and more WS with little risk of creating more SS. However, it is reasonable to expect that processing samples with increased time and/or speed in the impact dehuller would result in more SS (and more broken SS) than what we reported. As this is, to our knowledge, the first publication regarding sainfoin seed pod processing, a factorial experiment with different equipment settings was beyond the scope of this study. We reported *PE* metrics for each processing method which includes all three seed object classes IP, WS, and SS, but the results of the current study suggest that *DE* could be calculated by a two-stage processing method by first depodding with the belt thresher and then dehulling the resulting WS sample with the impact dehuller. There is a great need for additional follow-up studies regarding the appropriate processing machinery and machinery settings, and establishing the relationship between seed pod traits such as size and moisture content, and processing traits like *PE* and *DE*.

### Choosing the correct sample size

While the processing experiment shed some light on the nature of seed pod processing with many replications, the main goal was to determine the minimum detection limits between the difference of two single samples’ *PE* at any sample mass. Since it is much more convenient to evaluate many breeding lines on a single plant, single sample basis, we need an accurate estimate of the power of any comparison between two plants. In our analysis, we modeled two scenarios to determine the lower bounds of the detection limit between two samples with at least 80% power. We found that while sub-1g seed samples are far under-powered regarding the ability to discriminate between two samples’ *PE* centered on 0.5, it is possible to detect differences of around 0.20 with samples that are on the extremes of the distribution (say 0.95 and 0.75) at 1g (**Figure 10**). Our also results indicate that if we want to reliably detect a 0.25 absolute difference in *PE* between two sainfoin samples no matter where the two proportions lie on the binomial distribution, sample sizes should be at least 2g (**Figure 11**). If the *PE* values are closer to 0 or 1, smaller absolute differences down to 0.147 are reliably detected. Larger 4-5g samples afford reliable discrimination of smaller differences in *PE* with 80% power, but among the *PE* values we tested in our study, the lowest difference in *PE* was only 0.094 between two 5g samples. Detecting differences lower than this with sufficient power would require sample masses far greater than what we used for this study.

Historic sainfoin pod yields in our single plant field trials range from 2–75 grams per plant (data not shown). A 2g sample is a reasonable sample mass for greenhouse grown plants, and at that sample size, we can readily screen out lines with high vs low *PE*. Our pairwise analysis of the measured power for difference in *PE* between any two 2g samples reveals that 1,752 out of 4,950 comparisons had a statistical power above 0.8. Of these comparisons, the average effect size was 0.63 which indicates only medium-large *PE* spreads can be reliably discriminated at 2g.

Additionally, there are other constraints based on convenience to consider. We imaged processed samples using a large format platform and camera to capture the entirety of 5g samples. However, it may be convenient to image samples in a weigh boat or on a smaller platform in the processing pipeline where a 4-5g sample may be too crowded to allow accurate seed object detection. When making decisions such as these, researchers should consider the power cost associated with smaller sample sizes.

While we presented a theoretical power analysis based on the absolute difference in our *PE* proportions, it is also possible to conduct similar power analyses on penalized *PE*. Since *PE*_*SS*_ and *PE*_*WS*_ deviate from a binomial/multinomial distribution as they are linear combinations of random variables, one would need to estimate the variance using the delta method (Oehlert, 1992) or a simulation analysis and adjust the power calculation in Equation 10 accordingly. Such analysis is beyond the scope of the present investigation.

In breeding programs targeting new or underutilized crops, such as sainfoin, it is critical to ensure that phenotyping methods are not only innovative but also demonstrably capable of capturing the traits necessary to achieve defined breeding objectives. Unlike well-established crops with mature breeding pipelines, novel species often require the parallel development of reliable, scalable phenotyping tools that can meaningfully inform selection indices and advancement decisions. The findings presented here underscore the importance of method validation—unproven or noisy trait estimates can compromise selection accuracy, limit genetic gain, and obscure promising germplasm. By establishing the reliability and discriminative potential of phenotyping approaches early in the breeding process, programs can better align trait measurement with long-term goals for crop improvement. This is especially important in the context of exotic germplasm development, where broader trait exploration is common and breeding targets may span agronomic performance, nutritional value, and environmental adaptation. Ultimately, such rigor in trait measurement enhances the efficiency and impact of breeding strategies, supporting broader goals in food security, ecosystem resilience, and the development of climate-adapted crops.

## Conclusion

The present study developed and applied an image-based phenotyping pipeline for processing efficiency in sainfoin. First, we trained a Faster R-CNN model that successfully detected and classified intact pods (IP), whole seeds (WS), and split seeds (SS) with mAP75 of 0.9257. Second, we introduced a generalized metric, processing efficiency (*PE*), which effectively summarized the proportion of depodded and dehulled seeds after processing sainfoin pods. Third, our factorial analysis showed that processing method had the largest influence on *PE*, while the variety contributed additional but smaller effects [insert significant p-values or effect sizes if possible]. Fourth, power analysis indicated that sample sizes below 2g lacked sufficient power to reliably detect our target proportional difference in *PE* of 0.25 between two samples with 80% statistical power, underscoring the risks of under-sampling in breeding programs. Finally, by integrating deep learning–based object detection with classical statistical modeling, we demonstrated a scalable, accurate, and reproducible framework for high-throughput phenotyping of seed processing traits. Our findings establish minimum sample sizes for robust estimation of seed processing traits in sainfoin and highlight the potential of this approach to advance breeding selection indices and accelerate the improvement of existing sainfoin germplasm to establish it as a perennial legume grain crop.

## Data Availability

The image dataset and FasterRCNN model weights presented in the study are deposited in publicly available Zenodo repositories under accession numbers https://doi.org/10.5281/zenodo.8346923 and https://doi.org/10.5281/zenodo.8387982. All Python and R code used in this study are deposited in a public GitHub repository at https://github.com/BoMeyering/sainfoin_seed_RCNN.
